# Emotional Intelligence of Women Who Experience Domestic Violence

**DOI:** 10.1007/s11126-015-9368-0

**Published:** 2015-05-16

**Authors:** Konstantinos Tsirigotis, Joanna Łuczak

**Affiliations:** Department of Psychology, The Jan Kochanowski University in Kielce, Piotrków Trybunalski Branch, Słowackiego 114/118 str., 97-300 Piotrków Trybunalski, Poland

**Keywords:** Emotional intelligence, Domestic violence, Women

## Abstract

Violence in family constitutes serious social and psychological problem with harmful consequences leading, among others, to changes in emotional functioning of victim and, secondarily, also perpetrator. The aim of this study was to examine emotional intelligence of women experiencing domestic violence. INTE, i.e. Polish version of “Assessing Emotional Scale” by Schutte, was used to study two groups of women. Study (criterion) group included 40 women aged 23–47 years (mean age 35.28) using assistance of Crisis Intervention Centre due to experienced domestic violence. Reference (control) group was well-matched in terms of socio-demographic characteristics and consisted of 140 women not experiencing domestic violence. Study women experiencing domestic violence have significantly lower scores on all INTE indicators (general score, Factor I and Factor II). Women not experiencing domestic violence achieved significantly higher scores on Factor I than on Factor II. In this group all INTE components (general score, Factor I, Factor II) are positively correlated, whereas in group of women experiencing domestic violence there is no significant correlation between Factor I and Factor II and coefficients are lower. Emotional intelligence of study women experiencing domestic violence is lower than emotional intelligence of women not experiencing domestic violence. Their abilities and skills making up emotional intelligence are also less developed. The internal structure of emotional intelligence of study women experiencing domestic violence differs from emotional intelligence of women not experiencing domestic violence. It seems advisable to consider emotional intelligence in the process of providing women experiencing domestic violence with psychosocial help.

## Introduction

Violence in the family constitutes a serious social and psychological problem with harmful consequences for both individuals who experience violence and resort to it, leading, among others, to changes in the emotional functioning of the victim and, secondarily, also the perpetrator. Violence in the family may arise from emotionality disorders, personality disorders or psychotic disorders of the perpetrator, but it, certainly, also results from disturbances of relations between partners (regardless of the source of those disturbances). Violence in the family is, presumably, connected with disturbed emotionality, whereas emotional intelligence is associated with experienced emotionality.

The importance of emotional intelligence can be demonstrated, among others, by the idea thought up by researchers in the field of artificial intelligence to “add” emotions to computers in order to prioritize and direct their activity [1].

The construct of emotional intelligence has been formed as a result of an attempt at answering the question as to why some people are better than others at maintaining psychological wellbeing. For a long time, studies into intelligence were dominated by cognitive intelligence, although some researchers [cf. 2] drew attention to the fact that individuals having a high intelligence quotient (IQ) are not always efficient at coping with ordinary, everyday life and psychological tasks, while other individuals, with a lower IQ, come out very well at the same tasks. There is a view that it is differences in emotional intelligence that may be responsible for those discrepancies between cognitive intelligence and social functioning. Although Wechsler [3] focused on cognitive intelligence, he also mentioned non-cognitive aspects of general intelligence. In his definition of general intelligence, he mentions effective coping in one’s environment too, thus emphasizing the importance of ability to cope with and adapt to changing requirements of everyday life. Gardner [4] developed Wechsler’s idea and introduced two components of emotional intelligence: “intrapsychological abilities” and “interpersonal skills”. Based on that, Mayer and Salovay, who conducted many studies into emotional intelligence, developed the two Gardner’s components to come up with several ones [5, 6].

According to Salovay and Mayer’s model, emotional intelligence is a set of abilities and a subset of social intelligence that includes the following three categories of adaptive abilities: appraisal and expression of emotions, regulation of emotions and utilization of emotions in problem solving. The first category consists of components of appraisal and expression of own emotions and appraisal of emotions of others. The component of appraisal and expression of own emotions is further divided into two subcomponents, i.e.: verbal and non-verbal, while the component of appraisal of emotions of others is divided into subcomponents of non-verbal perception and empathy. The second category of emotional intelligence—regulation—includes components of regulation of emotions in self and regulation of emotions in others. The third category—utilization of emotions—incorporates components of flexible planning, creative thinking, redirected attention and motivation. Even though emotions are at the core of the model, it also includes social and cognitive functions connected with expression, regulation and utilization of emotions [1, 7]. Mayer et al. [8] further developed that model, but in the opinion of many authors, fundamental aspects of emotional intelligence proposed in the latest model are similar to those contained in the 1990 one [cf. 9].

Although some studies present concepts of ability emotional intelligence and trait emotional intelligence as mutually exclusive alternatives [e.g. 10], many authors believe that they both constitute important and mutually complementary dimensions of adaptive intellectual functioning [cf. 9].

Moods and emotions subtly but systematically affect some problem solving elements and strategies. Firstly, changing emotions may contribute to creating complex plans for the future. Secondly, positive emotions may modify organization of memories in such a way that cognitive material is better integrated and different ideas are seen as more associated with one another. Thirdly, emotions cause breaks in the work of complex systems as they “throw them off” a given level of operation and focus them on stronger needs.^1^ Finally, emotions and moods can be utilized in motivating and supporting the execution of complex mental tasks. Consequently, individuals who have developed abilities connected with emotional intelligence understand and express their own emotions, recognize emotions of others, regulate affect and utilize moods and emotions to motivate adaptive behaviours [1]. Authors wonder whether it is not yet another definition of a healthy, self-actualizing individual.

Moreover, authors notice relationships between emotional intelligence and health. According to them, an emotionally intelligent individual can be considered to be such that has achieved at least a certain form of positive mental health. Such individuals are aware of their own and others’ feelings. They are open to positive and negative aspects of internal experience, able to name them and communicate them when needed. Such awareness often leads to the effective regulation of one’s own emotions and emotions of others, hence contributing to wellbeing [1].

Other authors also report a positive impact of emotional intelligence on the life and psychological and social functioning of the individual. Better perception, understanding and managing of emotions by individuals of higher emotional intelligence may prevent the occurrence of non-adaptive emotional states associated with mood disorders and anxiety disorders. Studies proved that individuals of higher emotional intelligence have a tendency towards positive mood and are more capable of improving their mood after negative one [11, 12]. Generally speaking, higher emotional intelligence is connected with better psychophysical health [12].

Another researcher of emotional intelligence [2] also noticed a favourable impact of emotional intelligence, stating that emotionally intelligent individuals are competent at understanding themselves and others, in relations with others, as well as at adapting and coping in their environments. That, in turn, contributes to their ability to effectively cope with environmental requirements. To him, emotional intelligence is associated with direct functioning, while cognitive intelligence is connected with long-term strategic competence. In other words, emotional intelligence is process- rather than result-oriented [2, 6].

While emotional intelligence may have a favourable influence on the life and psychological and social functioning of the individual, another phenomenon, i.e. violence, exerts a rather negative influence.

Specialist literature offers many definitions of violence in the family. In Poland, a definition contained in the national programme of prevention of violence in the family is most commonly accepted. It describes violence as any action, intended and using an advantage of power, directed against a family member, which infringes his or her personal rights and interests, causing suffering and harm [13]. Another definition mentioned in Polish literature presents domestic violence as actions or glaring neglects on the part of a family member against the others, using an existing advantage of power or authority, or such an advantage created by circumstances, and causing harm or suffering to the victims, infringing their personal rights and, in particular, damaging their lives or (physical or mental) health [14].

Violence in the family may concern all its members; it can also be of the mutual nature. In the case of physical violence, however, perpetrators tend to be men [15]. The essence of domestic violence is the use of an advantage of power or authority in order to harm the other family members. Browne and Herbert distinguish among physical, psychological and sexual violence, drawing attention to its active or passive forms and intensity. Domestic violence victims experience anxiety, suffering, helplessness, dispiritedness and despair. Their bodies and psyches suffer acute injuries and are subject to the processes of damaging and protracted stress and threat [14].

The United Nations Declaration on the Elimination of Violence Against Women [16] defines violence against women taking place in the family in the following way: “Physical, sexual and psychological violence occurring in the family, including battering, sexual abuse of female children in the household, dowry-related violence, marital rape, female genital mutilation and other traditional practices harmful to women, non-spousal violence and violence related to exploitation”.

Research into the phenomenon of domestic violence most commonly focuses on identifying risk factors of its occurrence and diagnosing psychological traits of the functioning of its victims or perpetrators.

A majority (if not all) studies into emotional intelligence in connection with domestic violence have concerned mainly (if not exclusively) male perpetrators. An attempt at examining the relationship between emotional intelligence and violence experienced by women seems to be crucial for a more effective planning of preventive and therapeutic measures directed at that very group. Similar research, focused on the analysis of emotionality in families affected by violence, concerned the phenomenon of empathy described as a form of relation where one person experiences and shares emotions of another [17]. In a family where violence occurs, ability to empathize is disturbed. An individual who uses violence may not feel empathy and relation with others [18]. Deficits of empathy towards self occur in both victims and perpetrators of violence. A harmed person does not sympathize with himself or herself, which may contribute to repeated exposure to violence [17]. Disorders of emotions have very serious consequences for the parent–child relationship, too. A parent who has no contact with his or her emotions has disturbed empathy, may not be able to empathize with the child’s feelings and situation, may be aggressive or excessively demanding, which makes it difficult to build close ties and may increase violence used against the next generation [19].

It was observed that symptoms of violence in relationships occur as early as before entering into marriage [20]. It is worth mentioning that emotional intelligence may be a factor preventing psychological violence in relationships, which was confirmed by research results [20, 21].

As far as individuals experiencing domestic violence are concerned, it was established that they try to defend themselves applying seven universal (according to Lee Bowker) strategies: try to talk to the perpetrator, try to extract promises, try to nonviolently threaten the perpetrator, hide, use passive defence, avoidance and counterviolence. Moreover, literature mentions the so called humiliating strategies [14]. Probably, as a result of counterviolence and, certainly, as a result of experienced threat and anxiety (battered wife syndrome), they sometimes commit manslaughter [22].

As for their emotional intelligence, it was found that perpetrators of domestic violence have lower emotional intelligence than the general population, which seems to be associated with a stronger tendency towards violence. Furthermore, domestically violent men have general deficits of global social functioning, which results from the fact that they may not be aware of their emotions and have no insight into how their emotions are formed. It is important to notice that emotional intelligence deficits are connected with a tendency towards violence in both the population of violence perpetrators and the general population [6].

So far, studies into emotional intelligence in the aspect of domestic violence have concerned almost solely perpetrators of that violence. World literature offers almost no studies into emotional intelligence of women experiencing domestic violence.

The aim of this study has been to examine emotional intelligence of women experiencing domestic violence.

Therefore, the following hypotheses are assumed:

1. Emotional intelligence of study women experiencing domestic violence is lower than the emotional intelligence of women not experiencing domestic violence.
2. Their ability to use their emotions and their ability to recognize emotions are less developed.
3. Their ability to utilize emotions and the ability to recognize emotions are not correlated with each other.

## Methods

### Participants

The INTE was used to study two groups of women. The study (criterion) group included 40 women aged 23–47 years (mean age 35.28) using assistance of the Crisis Intervention Centre (CIC) due to experienced domestic violence. Women reported to CIC on their own initiative or were addressed to it by the interdisciplinary team for the prevention of domestic violence and all had “Blue Card”.^2^ The research was conducted by specialists (psychologists) at the start of the intervention, after informing women about the aim of the study and obtaining their consent to participate in the study. The reference (control) group was well-matched in terms of socio-demographic characteristics and consisted of 140 women not experiencing domestic violence.

### Materials

The tool to examine emotional intelligence was created by Schutte et al. [7]. Since then, the questionnaire has been used in many studies, but under different names: “Emotional Intelligence Scale” (EIS) [9, 23, 24], “Schutte Self-Report Inventory for Emotional Intelligence” (SSRI) [25] and “Schutte Emotional Intelligence Scale” (SEIS) [9, 26]. That has most probably resulted from the fact that the authors of the tool did not give it a name on its creation [7]! They only mentioned “emotional intelligence scale” [7, p. 175], although as a common rather than proper name. They first used the “Assessing Emotional Scale” (AES) name in later studies [9, 12]. The tool was translated from English into several languages: Hebrew, Polish, Swedish, Turkish [9]. This study applies the “Kwestionariusz Inteligencji Emocjonalnej INTE” (Emotional Intelligence Questionnaire INTE), i.e. the Polish version of AES, as adapted by Ciechanowicz, Jaworowska and Matczak [27]. The questionnaire is composed of 33 items on which the subject may take a position by choosing one of the five possible answers (the Likert type scale). Along with the general emotional intelligence score (sten score), the scale enables to receive scores on two factors: Factor I is ability to utilize emotions in order to support thinking and actions, while Factor II is ability to recognize emotions. Both the American and Polish versions are characterized by high reliability and validity [7, 27].

### Statistical Analysis

The statistical analysis of received scores applied descriptive methods and statistical inference methods. In order to describe the mean value for quantitative traits, the arithmetic mean (M) was calculated, while the standard deviation (SD) was assumed to be the dispersion measure. The conformity of distributions of quantitative traits with the normal distribution was assessed using the Shapiro–Wilk test. Due to the lack of conformity of distributions of dependent variables with the normal distribution, the statistical processing of received results used non-parametric statistics: the Mann–Whitney “U” test to examine inter-group differences and Kendall’s “tau” (*τ*) correlation coefficient to examine relationships between the studied variables. For all the analyses, the maximum acceptable type I error was assumed at *α* = 0.05. Asymptotic two-sided test probability *p* was calculated and *p* ≤ 0.05 was considered statistically significant. The statistical analyses were performed by means of the *Statistica PL 10.0 for Windows* statistical package [28].

## Results

Table 1 shows socio-demographic data of the studied groups; there are no differences in socio-demographic variables because, as mentioned in the Material and Method section, the reference group was well-matched to the study group in terms of those characteristics.

**Table 1:** Socio-demographic characteristics of studied groups

Variable	Violence		No violence	
	N	%	N	%
Age				
M ± SD	35.28 ± 1.5		35.15 ± 1.8	
Range	23–47		23–46	
Marital status				
Single	1	2.50	3	2.14
Non-formalized relationship	4	10.00	14	10.00
Married	29	72.50	102	72.86
Divorced	6	15.00	21	15.00
Education				
Primary	–	–	–	–
Vocational	21	52.50	73	52.14
Secondary	15	37.50	53.00	37.86
Higher	4	10.00	14	10.00
Socio-economic status				
Low	15	37.50	52	37.14
Medium	17	42.50	60	42.86
High	8	20.00	28	20.00
Place of residence				
Countryside	15	37.50	52	37.14
Small town	18	45.00	63	45.00
Big city	7	17.50	25	17.86

**Fig. 1:**
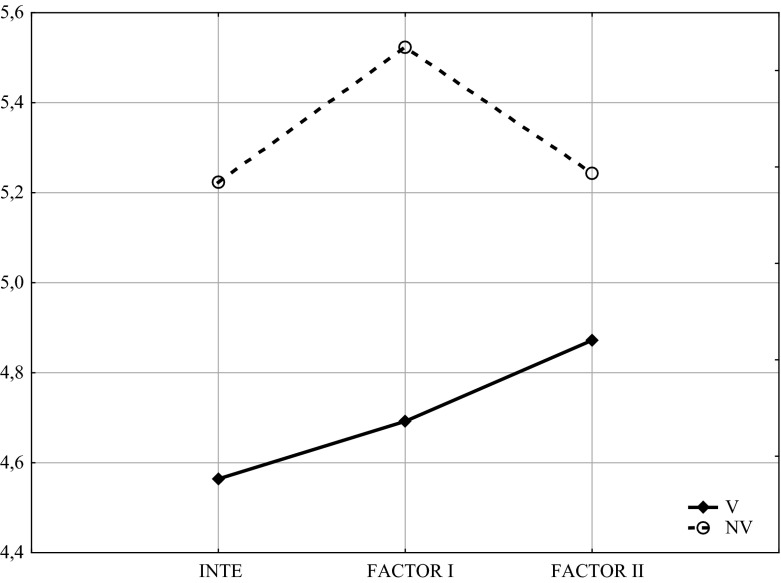
Comparisons of INTE sten scores of women experiencing (V) and not experiencing (NV) domestic violence

Table 2 and Fig. 1 present differences in scores achieved by the group of women experiencing domestic violence (the V group) and the group of women not experiencing domestic violence (the NV group); it can be observed that women experiencing domestic violence have significantly lower scores on all the INTE indicators (general score, Factor I and Factor II).

**Table 2:** Comparisons of INTE sten scores of women experiencing (V) and not experiencing (NV) domestic violence

Variables	V group			NV group			U	Significance
	Mean	St. Dev.	Ranks sum	Mean	St. Dev.	Ranks sum		*p*
INTE	4.564	1.518	3015	5.223	1.731	12,916	1884.5	0.003
FACTOR I	4.692	1.625	2719	5.523	1.754	13,212	1939.0	0.005
FACTOR II	4.871	1.657	3348	5.243	1.921	12,583	1910.0	0.004

Moreover, women not experiencing domestic violence achieved significantly higher scores (*p* = 0.001) on Factor I (ability to utilize emotions to support thinking and actions) than on Factor II (ability to recognize emotions); on the other hand, women experiencing domestic violence achieved non-significantly differing scores; there was a slight opposite tendency.: Factor II was a little higher than Factor I, although the difference was not statistically significant. At this point, it should be reminded that the general INTE score is not the sum of both the factors; moreover, the mentioned scores are standardized (sten scores).

**Fig. 2:**
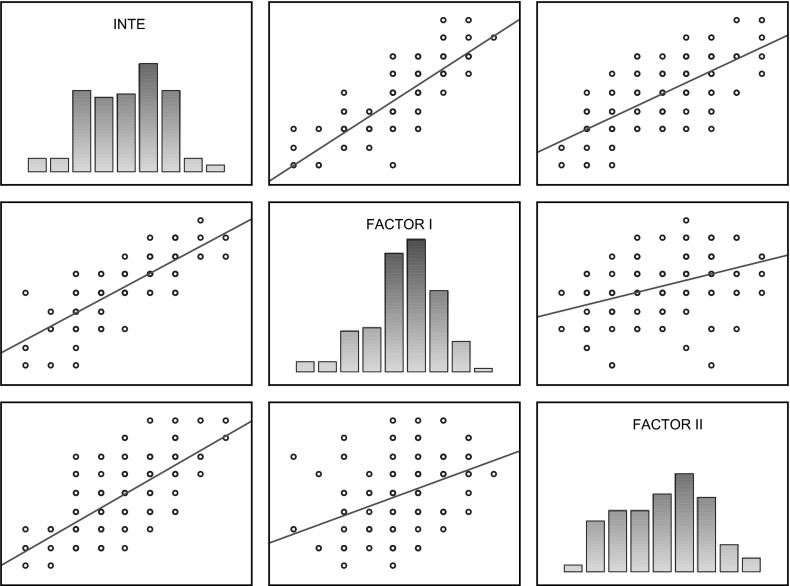
Scatterplot matrix of INTE scores of women not experiencing domestic violence

Table 3 shows coefficients of correlation between the variables studied by means of the INTE in the group of women not experiencing domestic violence; it can be observed that all the INTE components (general score, Factor I, Factor II) are positively correlated and coefficients are quite high in that group. The scatterplot matrix of those scores is presented in Fig. 2.

**Table 3:** Coefficients of correlation between INTE variables in the group of women not experiencing domestic violence

Variables	INTE	Factor I	Factor II
INTE		0.836 *p* < 0.001	0.736 *p* < 0.001
Factor I	0.836 *p* < 0.001		0.470 *p* < 0.001
Factor II	0.736 *p* < 0.001	0.470 *p* < 0.001

**Fig. 3:**
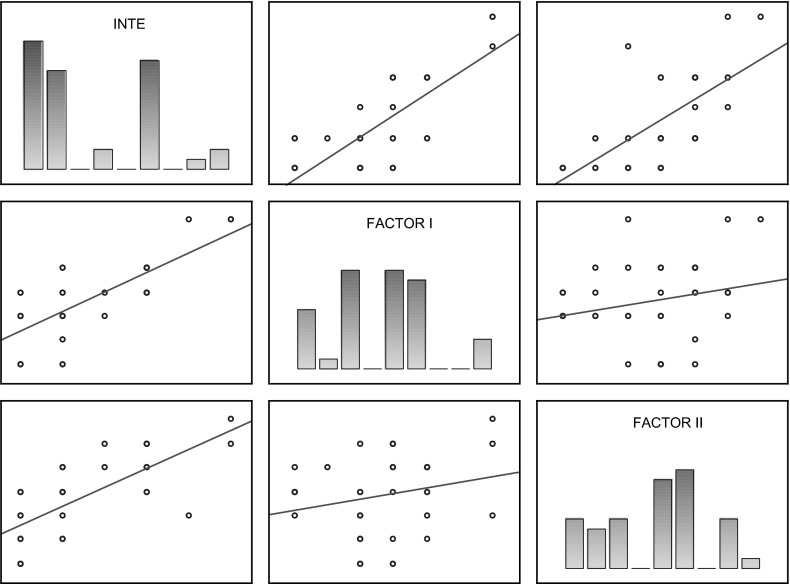
Scatterplot matrix of INTE scores of women experiencing domestic violence

Table 4 shows coefficients of correlation between specific INTE scales in the group of women experiencing domestic violence. In that group, coefficients are lower and, what is more, there is no significant correlation between Factor I and Factor II. The scatterplot matrix of those scores is presented in Fig. 3.

**Table 4:** Coefficients of correlation between INTE variables in the group of women experiencing domestic violence

Variables	INTE	Factor I	Factor II
INTE		0.575 *p* < 0.01	0.597 *p* < 0.01
FACTOR I	0.575 *p* < 0.01		0.138 ni.
FACTOR II	0.597 *p* < 0.01	0.138 ni.

## Discussion

In the discussion of results, it will be difficult to refer to results of other research in that field because the authors of this study have not found studies dedicated to that issue in available literature.

The presented research results indicate that abilities and skills making up emotional intelligence in women experiencing domestic violence are less developed/formed than in women not experiencing domestic violence. That can mean that women experiencing domestic violence are worse at recognizing emotions and can utilize them in their lives to a lower degree than women not experiencing domestic violence. Such a result is consistent with the observation that there is a relationship between lower emotional intelligence and worse psychosocial functioning [12].

Supposedly, irrespective of whether lower emotional intelligence is the cause or result of experienced domestic violence, it contributes neither to psychological functioning and wellbeing of the victim nor familial relations or relations between partners. Thus, it can be expected to be a factor interfering with or even disturbing relations between partners, which, combined with experienced domestic violence, can make the bad situation of the victim even worse.

Taking into account the fact that domestic violence perpetrators are also characterized by lower emotional intelligence [6], relations between such partners may be difficult at best and the relationship itself—stormy. Decreased abilities to recognize, name, constructively express and, in particular, utilize emotions in relations between partners and in life may lead to acts of aggression and victimization of women.

Differences in specific INTE factors in each group appear to be interesting.

Supposedly, women not experiencing domestic violence recognize emotions to a lower degree than utilize them in everyday life, i.e. they are better at utilizing than recognizing emotions. In turn, women experiencing domestic violence recognize and utilize emotions in resolving tasks and psychosocial functioning to a similar (low) degree. In a way, it is a developmental phenomenon: changes occurring in adulthood concern mainly abilities of a more complex nature [5, 11, 27].

The difference in relationships between specific dimensions of emotional intelligence in each group may be consistent with the above observations. As already mentioned, relationships between abilities making up emotional intelligence in the group of women experiencing domestic violence are weaker. Moreover, in the group of women not experiencing domestic violence, there are strong relationships between ability to recognize emotions and ability to utilize them; it can be assumed that recognizing emotions contributes to utilizing them and vice versa, through feedback mechanism, utilizing emotions contributes to learning how to correctly recognize them. On the other hand, in the group of women experiencing domestic violence, there are weaker relationships, in general, between abilities making up emotional intelligence. Moreover, in that group, there is no relationship (or it is very weak) between ability to recognize emotions and ability to utilize them to support thinking and actions; it may be one of the reasons for lower emotional intelligence of women experiencing domestic violence.

The above results, i.e. no differences in Factor I (ability to utilize emotions) and Factor II (ability to recognize emotions) as well as no relationships between them in the group of women experiencing domestic violence, may indicate different internal structures of emotional intelligence in those two groups. Although their ability to utilize emotions is better developed than their ability to recognize them, there are relationships between the abilities—they are correlated—in women not experiencing domestic violence; in other words, they are associated, despite differences, with each other. On the other hand, although those abilities are similarly (weakly) developed in women experiencing domestic violence, there are no relationships between them; in other words, although they do not differ, there are no relationships between them. That may suggest that the internal structure and cohesion of abilities and skills making up emotional intelligence are disturbed in women experiencing domestic violence.

At this point, a question ought to be asked about the cause and effect relationship: Is lower emotional intelligence in women experiencing domestic violence a cause or effect of experienced violence? One attempt at answering that question may lead to stating that abilities or skills making up emotional intelligence have been disturbed or damaged due to experienced domestic violence. Another attempted answer may indicate that disturbed or damaged abilities or skills making up emotional intelligence have been the cause of domestic violence as they made women choose such partners that use violence in general (including in the relationship), not noticing or ignoring earlier signals of danger; another possibility may be that those women fell victim to domestic violence as a result of disturbed relations between partners caused by low emotional intelligence. It seems that a definite answer to the above questions may be given by results of longitudinal studies.

Nevertheless, irrespective of the above, what can instil optimism is that emotional intelligence may be a factor preventing many pathological phenomena such as aggression and violence, also in relationships [10, 20, 21]. Therefore, it seems advisable to consider also that aspect of the psychological functioning of women experiencing domestic violence in actions aimed at providing them with psychosocial help.

## Conclusions

Recapitulating the above findings one can state that emotional intelligence of study women experiencing domestic violence is lower than emotional intelligence of women not experiencing domestic violence. Their abilities and skills making up emotional intelligence are also less developed. The internal structure of emotional intelligence of study women experiencing domestic violence differs from emotional intelligence of women not experiencing domestic violence. It seems advisable to consider emotional intelligence in the process of providing women experiencing domestic violence with psychosocial help.

### Limitations

The sample (V) size may be a possible limitation.

## Footnotes

1 As a matter of fact, for that reason, they were considered factors interfering with mental (intellectual) activity in the Western tradition of thinking about emotions.

2 “Blue Card” is an important part of the Polish system of intervention strategies against domestic violence existing in Poland since 1998 and is filled in at the intervention site in the presence of the perpetrator.
